# Expression of DSG1 and DSC1 are prognostic markers in anal carcinoma patients

**DOI:** 10.1038/bjc.2011.548

**Published:** 2012-02-14

**Authors:** M P Myklebust, Ø Fluge, H Immervoll, A Skarstein, L Balteskard, O Bruland, O Dahl

**Affiliations:** 1 Section of Oncology, Institute of Medicine, University of Bergen, Bergen 5021, Norway; 2 Department of Oncology and Medical Physics, Haukeland University Hospital, Bergen 5021, Norway; 3 Section of Pathology, The Gade Institute, University of Bergen, Bergen 5021, Norway; 4 Department of Pathology, Haukeland University Hospital, Bergen 5021, Norway; 5 Department of Surgical Sciences, University of Bergen, Bergen 5021, Norway; 6 Department of Surgery, Haukeland University Hospital, Bergen 5021, Norway; 7 Department of Oncology, University Hospital of Northern Norway, Tromsø 9038, Norway; 8 Center for Medical Genetics and Molecular Medicine, Haukeland University Hospital, Bergen 5021, Norway

**Keywords:** DSG1, DSC1, E-cadherin, anal carcinoma and survival

## Abstract

**Background:**

Our purpose was to investigate if dysregulation of cell adhesion molecules could be linked to prognosis in squamous cell carcinomas (SCCs) of the anal region.

**Methods:**

Protein expression of desmoglein-1 (DSG1), desmocollin-1 (DSC1) and E-cadherin was studied by immunohistochemistry in a cohort of 53 anal carcinoma patients treated by radiation alone or combined with 5-fluorouracil and mitomycin C.

**Results:**

Univariate analyses identified, among others, negative membranous DSG1 staining (*P*=0.009), negative cytoplasmic DSC1 staining (*P*=0.012) and negative DSG1 (membranous)+negative DSC1 (cytoplasmic) staining (*P*=0.004) to be associated with improved cancer-specific survival (CSS). On multivariate analyses positive DSG1 (membranous)+DSC1 (cytoplasmic) staining (HR 6.95, *P*=0.044), large tumour size and lymph node metastases (HR 6.44, *P*=0.004) and radiation without chemotherapy (HR 6.73 *P*=0.004) were associated with worse CSS. On univariate analysis, improved disease-free survival was associated with negative membranous staining of DSG1 (*P*=0.047), and negative DSG1 (membranous)+negative DSC1 (cytoplasmic) staining (*P*=0.025), among others.

**Conclusion:**

Membrane negativity for DSG1 and cytoplasmic negativity for DSC1 are favourable markers for CSS in SCCs of the anal region.

Cell adhesion is mediated through transmembrane molecules binding either to extracellular matrix or to adjacent cells. The dynamic interactions and signal transduction of these proteins modulate cell mobility, differentiation and proliferation (Thomas and Speight, 2001). Among the major cell–cell adhesion proteins are cadherins, members of the plakin family and the armadillo protein family (Green *et al*, 2010). Two important types of cell–cell junctions are the desmosomes and adherens junctions. The desmosome is a type of intercellular junction important in epithelial differentiation (Kottke *et al*, 2006), and the major members among the desmosomal cadherins are the desmogleins and the desmocollins (Garrod *et al*, 2002). The malignant phenotype with invasion and metastasis is facilitated by the loss of intercellular adhesion (Shinohara *et al*, 1998; Christofori, 2006; Kundu *et al*, 2008). Most important, reduced expression of the adherens junction glycoprotein E-cadherin has been linked to poor patient outcome in diverse carcinomas (Schipper *et al*, 1991; Umbas *et al*, 1994). E-cadherin downregulation is also a hallmark of the epithelial–mesenchymal transition (EMT) (Peinado *et al*, 2004), which is an important feature of tumour progression, possibly mediated through expression of its repressors Snail, Slug and Twist (Hajra *et al*, 2002; Yang *et al*, 2004; Peinado *et al*, 2007). In squamous cell carcinomas (SCC) of the head and neck, downregulation of both E-cadherin and desmosomal proteins were associated with decreased survival in univariate analysis, while only E-cadherin remained significant in multivariate analyses (Bosch *et al*, 2005).

Squamous cell carcinomas of the anal region comprise tumours arising from the squamous epithelium-lined distal anal canal or the skin within 5 cm from the anal margin. Sexually transmitted human papillomavirus (HPV) is the primary cause of cervical carcinomas, and HPV is also thought to be the most important aetiological factor for the development of anal cancer (Frisch *et al*, 1999; Daling *et al*, 2004). Different HPV subtypes affect the skin, the oropharyngeal or the anogenital mucosa, and some are oncogenic through expression of viral proteins such as E6 and E7 (zur Hausen, 2000). Anal cancer usually occurs in middle-aged individuals, with a female predominance (Clark *et al*, 2004). Anal cancer is mainly cured by chemoradiation, which includes radiation to a total dose of 46–60 Gy concomitant with combinations of 5-fluorouracil and either mitomycin C or cisplatin. This therapy regimen yields 5-year cancer-specific survival (CSS) of 70–80% (Ajani *et al*, 2008). Prognostic factors in anal carcinomas have been reviewed, and tumour stage and nodal status are the most important prognostic factors for patient outcome (Fenger, 2002; Lampejo *et al*, 2010). A consistent, prognostic biomarker has yet to be identified, although several have been proposed to be predictors of patient outcome.

To test the prognostic impact of the desmosomal components desmoglein-1 (DSG1) and desmocollin-1 (DSC1), and the adherens junction protein E-cadherin, we analysed 53 SCC specimens from the anal region, performing immunohistochemical staining on tissue microarray (TMA) slides. Our working hypothesis was that reduced expression of DSG1 and DSC1 in the membrane would be associated with poorer prognosis. E-cadherin was included as others have shown that reduced E-cadherin expression is significantly associated with reduced survival and increased risk of metastasis in other SCCs (Koseki *et al*, 1999; Dursun *et al*, 2007; Muller *et al*, 2008).

## Materials and methods

### Clinical data and tissue specimens

For construction of the TMA block, 53 samples of formalin-fixed and paraffin-embedded (FFPE) tumour tissue from anal carcinoma patients were used. The histological diagnosis was SCC for all 53, including 4 of cloacogenic type, 1 of basaloid type and 1 of unclassified epithelial type. All were treated with curative intent by radiation alone (*n*=8, combined with surgery in three), or radiation combined with concomitant FuMi chemotherapy (5-fluorouracil, 1000 mg m^–2^ days 1–4, and mitomycin C, 10 mg m^–2^ day 1, *n*=45, 15 of these had also surgery). Of 45 patients treated with concomitant chemotherapy, 28 patients received one course and 17 received two courses of chemotherapy. The median radiation dose of these 53 patients was 50 Gy. Radiation fields encompassed primary tumour (distal margin 3 cm), the groins (1.5 cm from pelvic brim or with 1 cm margin from manifest lymph nodes) and iliacal nodes with upper border at the promontorium. The fields were customised after CT-based treatment plans. Mean follow-up time was 6.9 years. Mean age at diagnosis was 64.1 years. Among the 53 patients, 18 died of anal cancer and 2 died from treatment complications. Progressive disease and relapse was encountered in 23 of 53 patients. Of these, 5 patients had progression of their primary tumour, 8 had distant relapses while 10 patients had a local relapse.

### TMA and immunohistochemistry

To analyse protein expression of DSG1, DSC1 and E-cadherin, a TMA including 53 patients with SCC of the anal region were constructed from archival FFPE tumour samples. All biopsies were sampled before start of therapy. The corresponding haematoxylin and eosin stains were assessed to confirm the diagnoses of these patients, and the areas of interest were marked on the slides. A ‘Manual Tissue Arrayer 1’ with a 1.0 mm diameter punch-set (Beecher Instruments, Silver Spring, MD, USA) was employed to take out one or two cores from each sample.

From the TMA-block, 5 *μ*m sections were cut with a microtome and mounted on slides. The sections were deparaffinised in xylene and rehydrated through graded ethanol to distilled water. For DSG1 and DSC1, antigen retrieval was performed in a kitchen pressure cooker, at 120 °C for 15 min in 0.05% citraconic anhydride solution, pH 7.4. After heat-induced retrieval, the sections were allowed to cool to room temperature before washing in distilled water and TBST (0.05 M Tris-HCl, 0.15 M NaCl, 0.05% Tween 20, pH 7.5). Sections were blocked for nonspecific binding with ‘Protein Block Serum-Free’ (Dako, Glostrup, Denmark) for 10 min preceding incubation with primary antibody for one hour at room temperature. E-cadherin was immunostained using a Dako Autostainer. Heat-induced epitope retrieval for E-cadherin was performed in TE-buffer pH 9.0 in a microwave oven, 9.5 min at 850 W, followed by 15 min at 400 W and incubation with primary antibody for 30 min at room temperature. The following antibodies were used: anti-DSC1 (DSC1-U100, PROGEN Biotechnik GmbH, Heidelberg, Germany) (Kurzen *et al*, 2003), 1 : 20; anti-DSG1-solution (DSG1-P124, PROGEN Biotechnik GmbH) (Kurzen *et al*, 2003), 1 : 2; anti-E-cadherin (NCH-38, Dako), 1 : 100; anti-MCM7 (ab-2360, Abcam, Cambridge, UK) 1 : 200. Primary antibodies were diluted in Antibody Diluent (S0809, Dako) and endogenous peroxidases were blocked with Dual Endogenous Enzyme Block (Dako). The EnVision+ Dual Link System-HRP (K4065, Dako) with DAB was used for detection according to the manufacturer's recommendations for all antibodies. Counterstaining was performed with haematoxylin (REAL Hematoxylin, S2020, Dako). Primary antibody was omitted in the negative controls. A panel of tissues with known reactivity was used as positive control, that is, normal anal mucosa, normal, carcinoma *in situ* and SCC of the cervix and SCC from the skin. The protocol for immunostaining and scoring of MCM7 is described elsewhere (Bruland *et al*, 2008). Digital images for scoring were obtained using a semi-automatical Leica DM6000 B microscope with a Leica DFC320 camera and the Leica Application Suite TMA 6000 version 2.5 software (Leica Microsystems GmbH, Wetzlar, Germany).

The results from the immunohistochemically stained slides were scored independently by two of the investigators. The localisation (membranous, cytoplasmic or nuclear) and intensity of the staining was recorded and given a score; negative or positive for DSG1 and DSC1. E-cadherin was recorded as membranous weak (0–1) or strong (2–3). The scoring of MCM7 is described in our previous publication (Bruland *et al*, 2008).

Owing to technical reasons, the number of patients available for survival analysis for each of the three markers varied. For DSC1 and E-cadherin one of the tissue cylinders detached and was lost for scoring during tissue processing, but all 53 were present and representative for DSG1.

### Statistics and ethics

The results from the immunohistochemical analyses of DSG1, DSC1 and E-cadherin were assessed in relation to anal CSS and disease-free survival (DFS) using the Kaplan–Meier method. Cancer-specific survival was calculated from the date of diagnosis to the date of death or date of last follow-up. Disease-free survival was calculated from the date of diagnosis to the date of relapse, progression of cancer disease or date of last follow-up. The log-rank method was used to compare survival between groups, with a *P*-value of <0.05 considered statistically significant in two-tailed tests. Pearson's *χ*^2^ test and Fisher's exact test were used for analysis of associations between DSG1 and DSC1 and gender, age, treatment, T- and N-stage and MCM7-index. Cox’ regression analysis was performed to assess independent prognostic factors for CSS, including the variables treatment (chemoradiotherapy *vs* radiotherapy alone), stage (T1–2N0 *vs* T3–4N+), DSG1 (membranous)+DSC1 (cytoplasmic) staining (both negative *vs* one or both positive) and MCM7 staining (index <140 *vs* index ⩾140). The SPSS 18.0 statistical package was used for the statistical testing (SPSS Inc., Chicago, IL, USA).

Patients gave written, informed consent to sampling of a separate biopsy for research purpose. The study was approved by the regional ethics committee and thus complies with regulations for clinical research in Norway.

## Results

### Immunohistochemistry

**Figure 1:**
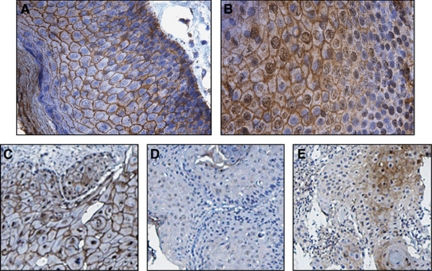
Immunohistochemical staining of normal anal mucosa and anal carcinomas (SCC) showing representative staining for DSG1 and DSC1. (**A**) DSG1 and (**B**) DSC1 staining in normal anal mucosa. (**C**) Positive membranous DSG1 in anal SCC. (**D**) Negative membranous DSG1 with weak perinuclear staining in anal SCC. (**E**) Positive cytoplasmic DSC1 in anal SCC.

In a cohort of 53 anal cancer patients, DSG1, DSC1 and additionally E-cadherin were analysed at the protein level by IHC. Desmoglein-1 showed specific staining of the cell membrane of the anal SCCs with varying intensity (Figure 1C). In normal anal mucosa, strong membranous staining and some diffuse cytoplasmic staining were observed. Staining was seen in all layers of the anal mucosa, with apparently more cytoplasmic staining of the basal cells (Figure 1A). For DSG1 membrane staining (Figure 1C), 31% of the tumours were scored positive. Perinuclear and cytoplasmic staining were also observed (Figure 1D), but no relation to clinical outcome could be found for this staining pattern. In normal anal mucosa, DSC1 showed strong membranous staining of the more differentiated layers. Cytoplasmic and nuclear staining was also observed. Towards the basal layer, the staining was mostly confined to the nucleus with some diffuse membranous staining (Figure 1B). Weak membrane and cytoplasmic staining was seen for DSC1 in the SCCs, but only cytoplasmic staining was statistically significant in relation to CSS (Figure 1E). The percentage of cytoplasmic DSC1-positive tumours was 30%. No statistically significant covariation between DSG1 membranous and DSC1 cytoplasmic staining was detected. E-cadherin showed strong, specific membrane staining and 67% of the tumours were scored as strongly positive. Representative examples of the immunohistochemistry staining patterns for DSG1 and DSC1 are shown in Figure 1.

### Survival analyses

No significant associations between DSG1 (membranous) and DSC1 (cytoplasmic) staining and age, gender, treatment, T- or N-stage were detected. A statistically significant association between DSG1 alone and MCM7 was observed (*P*=0.020). The association between DSG1 (membranous)+DSC1 (cytoplasmic) expression and high MCM7 expression was also statistical significant (*P*=0.030). The patient characteristics and histopathological variables are shown in Table 1.

**Figure 2:**
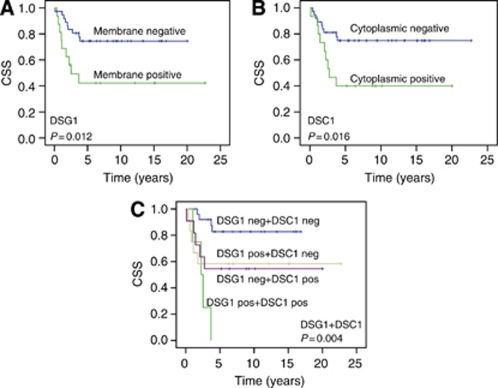
Univariate survival analyses according to the Kaplan-Meier method. Kaplan-Meier plots showing anal cancer specific survival (CSS) with *P*-values from log-rank test. (**A**) DSG1 membranous negative *vs* membranous postive. (**B**) DSC1 cytoplasmic negative *vs* cytoplasmic positive, and (**C**) DSG1 membranous +DSC1 cytoplasmic staining assessed as one variable.

Large tumour size and the presence of lymph node metastases were associated with a reduced 5-year CSS (*P*=0.002). Patients treated with chemotherapy in combination with radiation had a significantly better 5-year CSS than patients receiving only radiation with or without surgery (*P*=0.016). High expression of the MCM7 protein in tumour cells (index ⩾140) was associated with better CSS (*P*=0.005). For DSG1, negative membrane immunostaining was found to be favourable in this patient cohort. The 5-year CSS was 74% in patients with no membrane staining for DSG1, and 42% for those with positive membrane staining (log-rank test, *P*=0.012). Cytoplasmic negativity for DSC1 was also associated with better CSS (log-rank test, *P*=0.0012). The 5-year CSS was 72% among the patients with no DSC1 staining in the cytoplasm and 51% in those that were positive. Univariate analyses of the association of CSS and the different variables are shown in Supplementary Table 1. The Kaplan–Meier curves are shown in Figure 2. No statistic significant difference in CSS for membrane staining of E-cadherin was found (log-rank test, *P*=0.82, KM curve in Supplementary Figure 1). Regarding DFS, negative membranous DSG1 staining was found to be favourable (*P*=0.047). In the group with negative membranous staining, the 5-year DFS was 64%, compared with 44% for those with positive membranous staining. Improved 5-year DFS was also associated with small tumour size and no lymph node metastases (*P*=0.004) and high MCM7-index (*P*=0.035). DSC1 (cytoplasmic) staining was not found to have any statistically significant association with DFS. The results from the univariate analyses for DFS are shown in Supplementary Table 2 and Supplementary Figure 1.

**Figure 3:**
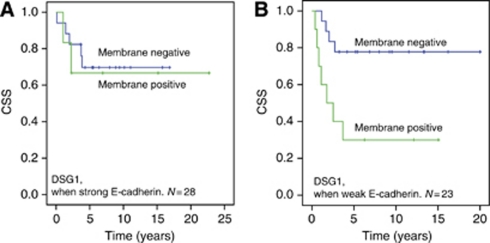
Cancer specific survival among the anal cancer patients when DSG1 membrane expression was stratified for E-cadherin expression. (**A**) DSG1 and cancer specific survival in patients with strong E-cadherin protein expression. (**B**) DSG1 and cancer specific survival in patients with weak E-cadherin expression. *P*-value=0.020 (pooled over strata).

To test whether expression of E-cadherin had any influence on the levels or signalling capabilities of DSG1 and DSC1, survival analyses were performed where the patients were stratified according to weak or strong E-cadherin expression. The results showed that for DSG1 membrane staining, the difference in survival between membrane negative and membrane positive was lower in subgroup with strong E-cadherin expression. For the patients with weak E-cadherin expression, the 5-year CSS was 78% for DSG1-negative *vs* 30% for DSG1-positive (Figure 3). In the subgroup of patients with strong E-cadherin expression, the 5-year survival was 70% for DSG1-negative *vs* 67% for DSG1-positive. The overall *P*-value is 0.020, meaning that the finding of better survival in patients with tumours negative for DSG1 membrane staining is still valid when we adjust for E-cadherin expression. No such difference between high and low levels of E-cadherin could be demonstrated for DFS for DSG1 membrane staining or DFS or CSS for DSC1 cytoplasmic staining.

When DSG1 (membrane) and DSC1 (cytoplasmic) were categorised as one variable, the outcome for the patients with tumours staining positive for both DSG1 membranous and DSC1 cytoplasmic protein was significantly worse than for the patients with no staining for DSG1 and DSC1. None of the patients positive for both DSG1 and DSC1 was alive after 5 years, while the 5-year CSS was 83% among the patients negative for both DSG1 and DSC1, and 58% and 55% for the patients positive for only DSG1 or DSC1, respectively (*P*=0.004) (Figure 2C). The 5-year DFS for DSG1+DSC1 was 68% for patients negative for both, 0% positive for both, and 58% and 55% for the patients positive for either DSG1 or DSC1, respectively (Supplementary Figure 1F).

The DSG1 (membranous)+DSC1(cytoplasmic) variable was merged to three categories instead of four in the Cox’ analysis as the DSG1-positive+DSC1-negative and DSG1-negative+DSC1-positive showed very similar curves in the survival analysis. The results from Cox’ multivariate analyses of CSS are shown in Table 2. Statistically significant predictors of reduced CSS survival were radiation without chemotherapy, large tumour size and lymph node metastases and positive staining for both DSG1 and DSC1. MCM7 did not reach statistical significance in the multivariate analysis.

## Discussion

We have studied the expression of the desmosomal proteins DSG1 and DSC1 in anal carcinoma and their relation to survival. To our knowledge, this is the first study to investigate the expression of these two desmosomal proteins and their associations to survival in SCC of the anal region. Membranous negativity for DSG1 was favourable for both 5-year CSS and DFS. Cytoplasmic negativity for DSC1 was favourable for 5-year CSS. Of the clinicopathological variables tested in this study, only MCM7 expression showed an association with the expression of DSG1. No such association between MCM7 and DSC1 expression was demonstrated. Multivariate survival analyses identified DSG1 and DSC1-status to be an independent prognostic variable for CSS in this cohort of anal carcinoma patients when expression of these two proteins was categorised as one variable. Treatment with radiation with or without chemotherapy and T- and N-stage of the tumours were also found to be independent prognostic variables. Treatment with chemoradiation is known to be favourable over radiation alone in anal cancer (Flam *et al*, 1996; Bartelink *et al*, 1997). Large tumour size and lymph node metastases are also well-known prognostic factors in anal carcinoma (Ajani *et al*, 2010; Ben-Josef *et al*, 2010). The MCM7 protein is associated with cell replication and this protein is essential for initiation of genome replication in eukaryotes. In a previous study, we demonstrated high expression of this protein to be favourable for the outcome in this cohort of anal carcinoma patients in a univariate analysis. Several other studies have identified this protein as a prognostic marker for different carcinomas (Li *et al*, 2005; Laitinen *et al*, 2008; Nishihara *et al*, 2008; Tamura *et al*, 2010; Ota *et al*, 2011). MCM7 protein expression was not identified as an independent prognostic variable in the present Cox’ analysis, although high expression of this marker was statistically significant associated with survival in univariate survival analysis. Thus, DSG1 (membranous)+DSC1 (cytoplasmic) seems to be a stronger predictor of survival than MCM7 in this cohort.

Our finding of highest survival rates among patients with negative protein expression for the two desmosomal cadherins was partly contradictory to other studies, which have implicated decreased expression of these cell adhesion molecules to facilitate metastatic and aggressive tumour behaviour and poorer prognosis in other squamous carcinomas and cell lines (Harada *et al*, 1996; Shinohara *et al*, 1998; Tada *et al*, 2000; Wong *et al*, 2008). Other studies have suggested that the organisation of the desmosomes may be changed during carcinogenesis. Tumours and metastases from head and neck cancers have been shown to harbour a heterogeneous pattern of desmosomes (Bosch *et al*, 2005). Bosch *et al* demonstrated that desmosomes of the tumours and metastases were generally fewer and reduced in size compared with normal tissue. Some endocytosed desmosomes were also recorded. E-cadherin and desmoglein levels were reduced in tumours *vs* normal tissue, but some staining persisted even in late stage tumours and metastases. From the results of this study, we cannot conclude whether the desmosomes positively stained for DSG1 and/or DSC1 are correctly assembled or not. It could be that some of the desmosomes with positive immunostaining actually represents non-functional desmosomes or desmosomes with incorrect assembly of its constituents.

However, there are several studies that present results that challenge the hypothesis that reduced expression of the desmosomal cadherins leads to a more aggressive and metastatic tumour phenotype. In skin carcinomas, no correlation between DSG1 and DSC1–3 expression and aggressive tumour behaviour could be demonstrated (Kurzen *et al*, 2003). Desmoglein-2, one of the other isoforms of desmoglein, has been shown to be upregulated and correlate with increased risk of metastasis in SCC of the skin (Kurzen *et al*, 2003; Brennan and Mahoney, 2009). Thus, it seems that several studies find the number of desmosomal cadherins to be reduced in premalignant and tumour tissue compared with normal tissue. Currently, the expression of desmosomal cadherins in SCC tumours is not fully clarified.

Desmoglein-1 and DSC1 are transmembrane proteins and serve as anchors between adjacent cells, but during the last decade it has become apparent that they may as well be involved in signalling governing epithelial morphogenesis (Getsios *et al*, 2009) and apoptosis (Dusek *et al*, 2006). The cytoplasmic tail of DSG1 and DSC1 is a binding site for the plakophilins 1–3 and plakoglobin of the armadillo family, reviewed in (Schmidt and Jager, 2005). Plakophilins 1–3 are implicated to have a dual role in cancerogenesis (Wolf and Hatzfeld, 2010). In the desmosome, they bind to DSG1 and DSC1 and may thereby act as tumour suppressors by stabilising intercellular adhesion. However, the cytoplasmic pool could have an oncogenic role by stimulating translation and proliferation by binding to the eIF4A initiation factor (Wolf *et al*, 2010). Plakoglobin may also act as an oncogene as it is a strong activator of the *MYC* promoter and an inducer of the anti-apototic *BCL2* (Hakimelahi *et al*, 2000; Kolligs *et al*, 2000), and it has also been implicated to work through the Wnt/*β*-catenin pathway (Chidgey and Dawson, 2007). The presence of plakoglobin may stabilise cytoplasmic *β*-catenin and allow enhanced translocation of *β*-catenin to the nucleus. Binding of plakoglobin could also displace *β*-catenin from the adherens junctions, again leading to increased levels of *β*-catenin in the nucleus. However, a tumour-suppressor role of plakoglobin has also been suggested because it can bind the transcriptional activator TCF-4 (Miravet *et al*, 2002) and thereby inhibit transcription of Wnt/*β*-catenin-responsive genes.

The above mentioned findings suggest that the function of DSG1 and DSC1 is more diverse than only being a rigid anchor and exert cell–cell contact. A deregulation of these proteins may therefore have several and unexpected effects in SCC. The localisation in different cell compartments may be of importance for their function. Our finding that the effect of membranous DSG1 staining on CSS varies with E-cadherin levels in the tumours is difficult to explain from the limited knowledge on the role of desmosomes in cancers (reviewed in Chidgey and Dawson, 2007; Dusek and Attardi, 2011). Loss of E-cadherin expression and dissolution of desmosomes are characteristics of EMT, which features tumour progression. We could not demonstrate any significant association between E-cadherin and survival in our cohort. However, it seems that the effect of DSG1 had less influence on survival when E-cadherin is highly expressed. High levels E-cadherin may mask the effects of membranous DSG1 loss. The loss of E-cadherin and membranous DSG1 expression could be explained EMT, but the finding of highest CSS in this subgroup of patients is difficult to explain from an EMT-perspective. The role of E-cadherin in correlation to DSG1 needs to be addressed in a larger material as there are few events (deaths) in each group in our material when we stratify for E-cadherin expression. Another important point when discussing cell migration and cell adhesion is that cell migration may happen in two different ways; single cell migration and collective (group) cell migration (Friedl and Gilmour, 2009). The decreased survival of the patients with apparently higher intercellular adhesion, for example, positive expression of DSG1 may be explained by increased collective migration of cells in these patients. Increased cell–cell contacts may enable groups of cancer cells to detach and migrate as a group of cells with intact cell–cell contacts between the migrating cells, thus providing a growth advantage (Collins *et al*, 1990).

Anal carcinoma is a rare type of cancer, and it is thus difficult to collect large series of tumours from this disease. In cervix, a tissue type that resembles the anal mucosa, DSG1 protein expression has been shown to be reduced during progression of squamous intraepithelial lesions (SILs) (de Boer *et al*, 1999; Alazawi *et al*, 2003). To our knowledge, no studies on DSG1 and DSC1 expression in cervical tumours in relation to survival have been published so far. Like SCC of the anal canal, SCC of the cervix is HPV-associated, and the two types of cancer have pre-malignant stages (ASIL and SIL). The common features of these two SCCs, calls for further analyses on desmosomal cadherins also in cervical carcinomas.

In conclusion, loss of DSG1 and DSC1 protein expression were significantly associated with better CSS in our cohort of anal SCCs, both when we studied the expression of the two separately, and more pronounced when assessed together.

## Figures and Tables

**Table 1:** Proportion of cases (%) for different clinical variables, according to DSG1 expression (panel A, 53 patients), DSC1 protein expression (panel B, 52 patients) and DSG1+DSC1 as one variable (panel C, 52 patients)

	**DSG1 membranous expression**		
**Panel A**	**Membr. neg *n*=37**	**Membr. pos *n*=16**	***P*-value**
Gender (*n*=53, female, %)	73.0	50.0	NS^a^
Age (*n*=53, <64.1 years)	51.4	31.3	NS^a^
Radiation with FuMi chemotherapy (*n*=53, %)^b^	86.8	81.3	NS^c^
TN-stage (*n*=52, T3–4 N0 and T_any_N+, %)	43.2	62.5	NS^a^
DSC1 cytopl. (*n*=52, pos, %)	30.6	25.0	NS^c^
MCM7 (*n*=52, index ⩾140, %)	75.0	31.3	0.003^a^

	**DSC1 cytoplasmic expression**		
**Panel B**	**Cytopl. neg *n*=37**	**Cytopl. pos *n*=15**	***P*-value**
Gender (*n*=52, female, %)	64.9	66.7	NS^a^
Age (*n*=52, <64.1 years)	40.5	60.0	NS^a^
Radiation with FuMi chemotherapy (*n*=52, %)^b^	86.5	86.7	NS^c^
TN-stage (*n*=52, T3–4 N0 and T_any_N+, %)	48.6	53.3	NS^a^
DSG1 membr. (*n*=52, pos, %)	32.4	26.7	NS^c^
MCM7 (*n*=51, index ⩾140, %)	64.9	57.1	NS^a^

	**DSG1(membr.)+DSC1 (cytopl.) expression**				
**Panel C**	**Both neg *n*=25**	**DSG1 pos DSC1 neg *n*=12**	**DSG1 neg DSC1 pos *n*=11**	**Both pos *n*=4**	***P*-value**
Gender (*n*=52, female, %)	76.0	41.7	63.6	75.0	NS^c^
Age (*n*=52, <64.1 years)	48.0	25.0	63.6.0	50.0	NS^a^
Radiation with FuMi chemotherapy (*n*=52)^b^	88.0	83.3	90.9	75.0	NS^c^
TN-stage (*n*=52, T3–4 N0 and T_any_N+)	44.0	58.3	45.5	75.0	NS^c^
MCM7 (*n*=51, index ⩾140, %)	84.0	25.0	60.0	50.0	0.003^c^

Abbreviations: cytopl.=cytoplasmic; DSC1= desmocollin-1; DSG1=desmoglein-1; membr.=membranous; neg=negative; NS=not significant (*P* >0.05); pos=positive; SCC=squamous cell carcinoma.

a *P*-values from *χ*^2^ statistics.

b Among 45 patients treated with radiation combined with FuMi chemotherapy, 15 patients had additional surgery, 8 patients had radiation with or without surgery, but no chemotherapy.

c Fisher's exact test.

Analyses include patients with SCC of the anal region treated with radiation or radiation with FuMi chemotherapy.

**Table 2:** Independent predictors for cancer-specific survival in 53 patients according to Cox’ regression model using death from anal cancer as endpoint

**Variable**	**Hazard ratio (95% CI)**	***P*-value**
*Treatment*		0.004
Radiation and FuMi±surgery	1	
Radiation±surgery, no chemotherapy	6.73 (1.86–24.35)	
		
*T- and N-stage*		0.004
T1–2 N0	1	
T3–4 N0 and T_any_ N+	6.44 (1.82–22.78)	
		
*DSG1 (membr.) + DSC1 (cytopl.) staining*		0.044
DSG1 neg+DSC1 neg	1	
DSG1+DSC1 different	4.77 (1.19–19.15)	
DSG1 pos+DSC1 pos	6.95 (1.38–34.94)	
		
*MCM7 staining-index*		NS
<140	1	
⩾140	0.39 (0.12–1.23)	

Abbreviations: CI=confidence interval; DSC1= desmocollin-1; DSG1=desmoglein-1; membr.=membranous; cytopl.=cytoplasmic; neg=negative; NS=not significant (*P*>0.05); pos=positive.

Cox analysis was based on 53 patients stained for both DSG1 and DSC1. One patient out of 53 had missing values for DSC1 and one for MCM7, these were not included in the analyses.
