# Supplementary figures and images for: Expression of DSG1 and DSC1 are prognostic markers in anal carcinoma patients
Source: Br J Cancer. 2012 Feb 14;106(4):756–62. doi: 10.1038/bjc.2011.548 (PMC3322941; doi:10.1038/bjc.2011.548)

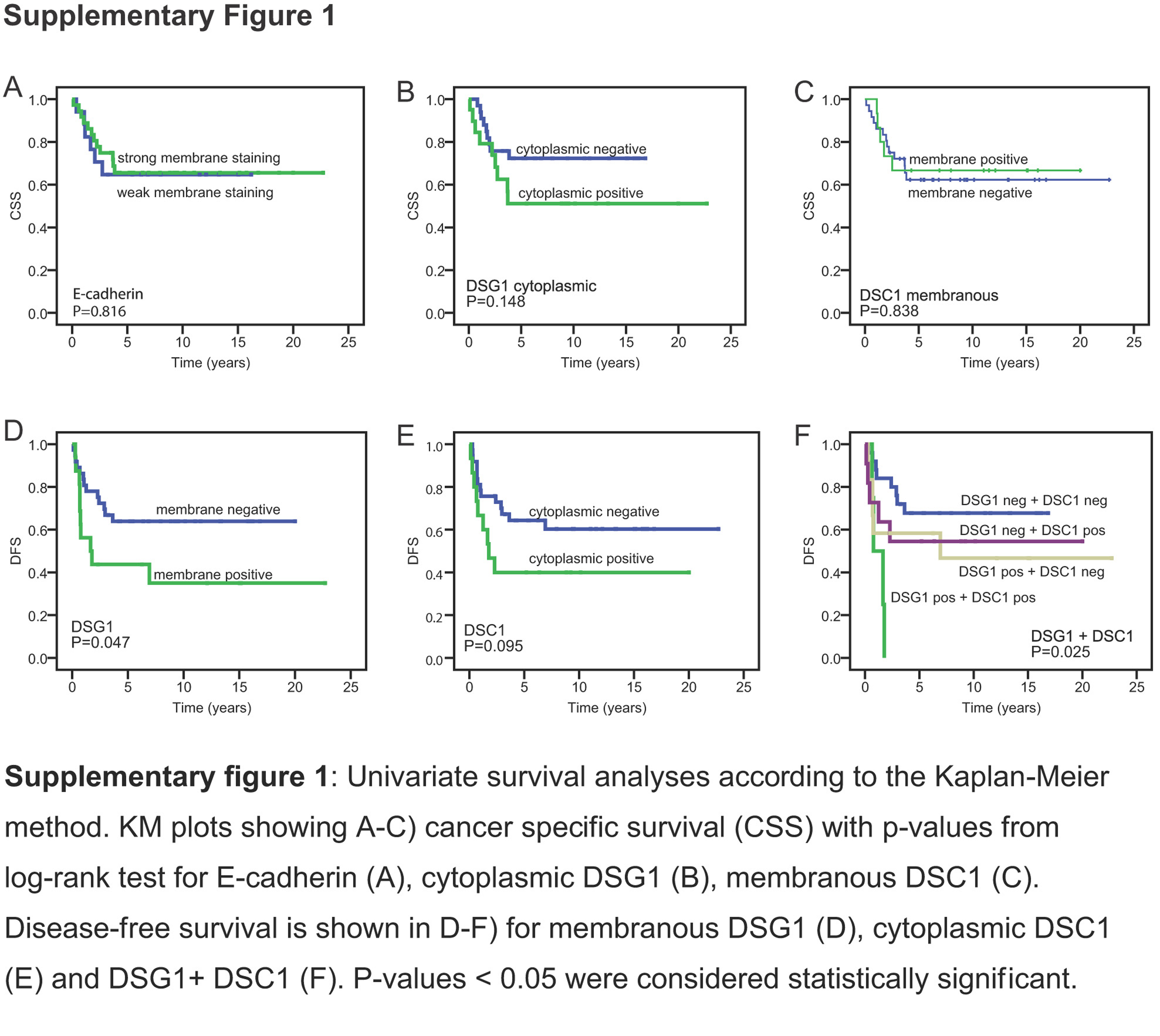

Supplement: Supplementary Figure 1 [file bjc2011548x1.tif]
