# Supplementary material for: Expression of DSG1 and DSC1 are prognostic markers in anal carcinoma patients
Source: Br J Cancer. 2012 Feb 14;106(4):756–62. doi: 10.1038/bjc.2011.548 (PMC3322941; doi:10.1038/bjc.2011.548)
Supplement: Supplementary Table 2 [file bjc2011548x3.doc]

**Supplementary Table 2**: Univariate associations of variables with disease-free survival (DFS) in 53 anal carcinoma patients treated with radiation or radiation with FuMi chemotherapy*.* P-values <0.05 were considered statistically significant.

| **Variable** | **n** | **5-year DFS (%)** | **s.e.**  **(%)** | **P** |
| --- | --- | --- | --- | --- |
| *Gender* |  |  |  | *NS* |
| Women | 35 | 55 | 9 |  |
| Men | 18 | 61 | 12 |
| *Age in years at diagnosis* |  |  |  | NS |
| <64.1 | 24 | 58 | 19 |  |
| ≥64.1 | 29 | 57 | 10 |  |
| *T- and N-stage* |  |  |  | *0.004* |
| T_1-2_N_0_ | 27 | 77 | 10 |  |
| T_3-4_N_0_ and T_any_N_0_ | 26 | 38 | 10 |
| *Treatment* |  |  |  | *NS* |
| Radiation ± surgery, no chemother. | 8 | 30 | 18 |  |
| Radiation with FuMi ± surgery | 45 | 62 | 7 |
| *DSG1 membranous* |  |  |  | *0.047* |
| Negative | 37 | 64 | 8 |  |
| Positive | 16 | 44 | 12 |
| *DSG1 cytoplasmic* |  |  |  | *NS* |
| Negative | 33 | 64 | 8 |  |
| Positive | 20 | 47 | 12 |
| *DSG1 nuclear* |  |  |  | NS |
| Negative | 28 | 55 | 10 |  |
| Positive | 25 | 60 | 10 |  |
| *DSC1 cytoplasmic* |  |  |  | *0.095NS* |
| Negative | 37 | 64 | 8 |  |
| Positive | 15 | 40 | 13 |
| *DSC1 membranous* |  |  |  | *NS* |
| Negative | 36 | 55 | 8 |  |
| Positive | 15 | 60 | 13 |
| *DSG1(membr.) + DSC1(cytopl.) staining* |  |  |  | *0.025* |
| DSG1 neg + DSC1 neg | 25 | 68 | 9 |  |
| DSG1 pos + DSC1 pos | 4 | 0 | 0 |
| DSG1 neg + DSC1 pos | 11 | 55 | 15 |
| DSG1 pos + DSC1 neg | 12 | 58 | 15 |
| *E-cadherin(membr)* |  |  |  | *NS* |
| Weak | 17 | 53 | 12 |  |
| Strong | 36 | 61 | 9 |
| *MCM7 staining (Bruland et al 2008)* |  |  |  | *0.035* |
| Index <140 | 20 | 43 | 11 |  |
| Index ≥140 | 32 | 65 | 9 |
